# Process Phenomena and Material Properties in Selective Laser Sintering of Polymers: A Review

**DOI:** 10.3390/ma15010183

**Published:** 2021-12-27

**Authors:** Federico Lupone, Elisa Padovano, Francesco Casamento, Claudio Badini

**Affiliations:** Department of Applied Science and Technology, Politecnico di Torino, Corso Duca degli Abruzzi 24, 10129 Torino, Italy; federico.lupone@polito.it (F.L.); francesco.casamento@polito.it (F.C.); claudio.badini@polito.it (C.B.)

**Keywords:** selective laser sintering, polymers, additive manufacturing

## Abstract

Selective laser sintering (SLS) is a powder bed fusion technology that uses a laser source to melt selected regions of a polymer powder bed based on 3D model data. Components with complex geometry are then obtained using a layer-by-layer strategy. This additive manufacturing technology is a very complex process in which various multiphysical phenomena and different mechanisms occur and greatly influence both the quality and performance of printed parts. This review describes the physical phenomena involved in the SLS process such as powder spreading, the interaction between laser beam and powder bed, polymer melting, coalescence of fused powder and its densification, and polymer crystallization. Moreover, the main characterization approaches that can be useful to investigate the starting material properties are reported and discussed.

## 1. Introduction

Powder bed fusion (PBF) processes are a widely adopted family of additive manufacturing (AM) technologies capable of producing three-dimensional (3D) parts showing unprecedented geometric complexity and good mechanical properties. These processes are based on a layer-by-layer deposition of powder particles; a laser beam is then applied to melt the powder in a locally restricted area of the current layer according to a digitally programmed design. Due to its build accuracy, high productivity for customized and complex-shaped objects, and good mechanical properties, selective laser sintering (SLS) of plastics shows an excellent capability to manufacture end products for most industrial applications. Polymer and composites parts with complex geometry can be fabricated without the use of supports or molds, making SLS a cost-effective alternative to other additive technologies (i.e., fused deposition modeling or stereolithography), or more conventional ones such as injection molding [1]. These advantages have encouraged the use of SLS in several contexts, from prototypes to the fabrication of spare parts and small series in automotive and aerospace fields, to customized prosthesis for biomedical applications [1]. Production tooling such as sacrificial molds and patterns for investment casting of complex metal parts has been also manufactured by using amorphous polymers such as polystyrene (PrimeCast^®^ powders from EOS Gmbh) and high-impact polystyrene [2,3]. In addition, the growing adoption of polymer composites, mainly based on a polyamide 12 matrix reinforced with carbon or glass fillers, has opened up new applications in which a light weight and functionality are required [4,5].

**Figure 1:**
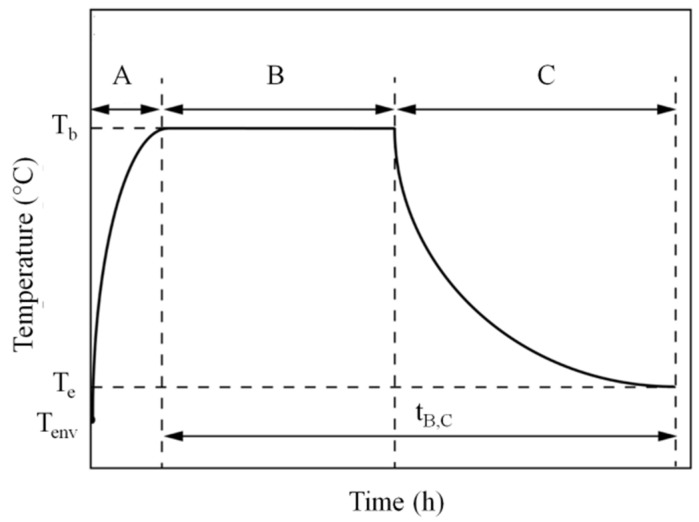
Schematic representation of the temperature profile of a standard SLS process. Adapted from [6].

The SLS process is essentially constituted of three main phases: the warm-up, build, and cooling stages. A typical temperature profile for the SLS process is shown in Figure 1.

In the warm-up stage (Figure 1A), the powder bed is preheated to a predefined temperature, referred to as the powder bed temperature (T_b_), which is maintained during the entire build process. The energy needed to reach this isothermal condition is usually achieved through continuous IR lamp irradiation and/or resistance heaters. In the build stage (Figure 1B), a new layer of powder is evenly spread onto the building platform by the recoating system. A blade or a rotating roller is usually adopted in commercial SLS machines, although noncontact powder-deposition systems based on vibratory nozzles or patterning drums were studied for multimaterial fabrication of parts [7,8,9]. A CO_2_ laser placed above the building chamber locally adds the extra energy needed to selectively melt the polymer grains on the surface of the powder bed along a “computer-controlled” pattern by means of a scanning mirror. After laser exposure, the melted powders coalesce at temperatures higher than the polymer melting point, and then slowly solidify upon cooling to the bed temperature (T_b_), thus ensuring the bonding between adjacent particles. The main driving force for particle coalescence is the viscous flow of the polymer melt (which is determined by the temperature-dependent viscosity), while the solidification is driven by the heat loss within the powder bed. These three sub-steps or “sub-processes” of (1) powder recoating, (2) laser energy input or “powder melting”, and (3) material consolidation occur during very short periods of time, between 35 s and 40 s depending on the SLS machine [6,10]. These substeps are sequentially repeated until the part is finished. Finally, in the cooling stage (Figure 1C), the sintered part, along with the entire build chamber, is gradually and evenly cooled down under homogeneous conditions until room temperature (T_e_) is reached [11,12].

Nowadays, a progressive shift of AM technology from fabrication of prototypes towards the production of end-use parts is taking place. Accordingly, the quality standards have become more stringent, and the physical and mechanical properties of the printed components have to meet in-service loading and operational requirements that are comparable to those of parts obtained by traditional manufacturing [13]. Recent advancements in SLS machines (e.g., improved laser and heating systems, uniform temperature distribution in the build area) have increased the maturity of this technology, thus surpassing some initial limitations [14]. However, further improvements are needed to achieve the industrial specifications in terms of component functionality, printing accuracy, process consistency, and reliability. For example, the residual porosity and the quality of the interfaces between adjacent layers (they are unavoidable in this process without affecting the conventional ones) strongly influence the ductility and the anisotropic behaviour of the printed parts [15,16]. Another issue concerns the thermal distortion, resulting in curling and warping of fabricated parts [17]. Therefore, to obtain components that achieve industrial standards, the three previously mentioned SLS subprocesses and their interactions must be further investigated.

In spite of the apparent simplicity of the SLS technique, the physical phenomena involved in the process are various and complex: powder spreading, interaction between the laser beam and powder bed, polymer melting, coalescence of fused powder and its densification, polymer crystallization, and shrinkage. Furthermore, these phenomena span over different scales of time and space. For example, considering the energy input supplied by the laser or “melting” substep, several issues concerning how the laser interacts with the powder bed, how the polymer melts, and how the thermophysical properties of the melting pools change over time play a critical role in determining the density and the microstructure of the final objects [15,18]. Furthermore, the quality of the sintered parts is strongly affected by various process parameters and material-specific characteristics: diameter of the laser beam, laser powder, scanning speed, hatching distance (i.e., spacing between adjacent laser scans), layer thickness, powder spreading method (i.e., roller or blade), bed temperature, type of polymer, powder size distribution, etc. [11,12].

This leads to the conclusion that polymer SLS is a very complex multiphysical process, and different aspects need to be addressed for a thorough understanding of the transient conditions of the powder grains and subsequent material consolidation. The present review aims at describing the physical phenomena involved in the SLS process and the characterization approaches useful to investigate the relevant material properties. Experimental analysis, theoretical models, and process modeling are reviewed in order to give a global view of the current knowledge of the technology, and to provide insights on the relationship between powders properties, process physics, and final part structure. This review could favor the adoption of improved processing strategies for existing thermoplastic polymers to reduce process interruptions and defective parts (e.g., negative effects such as warpage, curling, or porosity). Moreover, the discussion could also provide guidelines for the expansion of the material palette for polymer SLS.

## 2. Selective Laser Sintering Process

As previously mentioned, different physical phenomena are involved during polymer SLS component fabrication. These phenomena can be defined as a function of their characteristic temporal regimes:

- Laser motion and irradiation (10^2^ μs);
- Thermal diffusion (10^2^ ms);
- Polymer viscous flow and particle coalescence (10^1^ s);
- Powder spreading (1–10^1^ s);
- Solidification / crystallization (10^1^ min).

**Figure 2:**
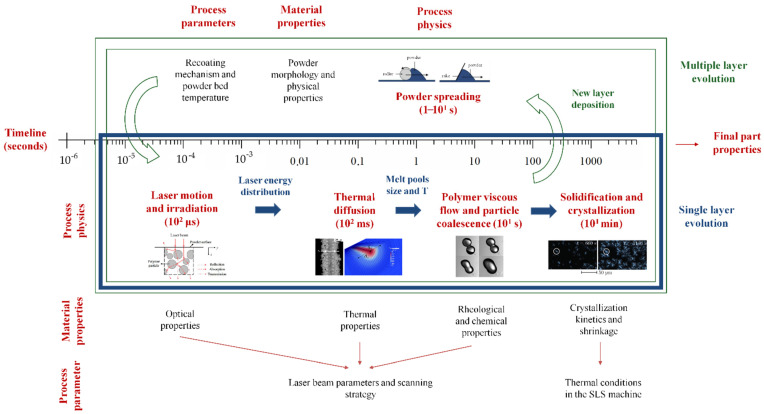
Timescale of the different physical phenomena involved in the SLS process, powder requirements for effective sintering, and relevant process parameters.

Figure 2 schematically illustrates these mechanisms along with their timescale, the properties of the polymer powders, and the process parameters that are highly significant for the macroscopic characteristics of the sintered objects.

The solid filled and hollow shapes (arrows and rectangles) highlight the production of a single layer and the fabrication of multiple-layer parts, respectively. The horizontal timeline describes the evolution of a single layer that moves down in z-direction during the running sintering process. The circle path illustrates what occurs when a powder layer is deposited and then the laser is applied; these steps are repeated layer-by-layer until the end of the printing job. This scheme also outlines the relationship between the physical phenomena involved in polymer SLS, the properties of the raw materials, and process parameters. [19,20]. The latter take into account the properties of the laser source, the laser energy input, and other machine-specific characteristics such as the heating system of the build chamber and the recoating mechanism employed. The starting powders suitable to be processed by SLS need to be characterized in terms of optical, thermal, rheological, and physical properties. A detailed investigation of the SLS process by considering the influence of different parameters can be useful to obtain preliminary selection criteria for the evaluation of a new material.

### 2.1. Powder Spreading (1–10^1^ s)

The rapid evolution of powder bed fusion technologies and the increasing applications at industrial scale make necessary both the development of powdered materials suitable to be processed with this technology, and the optimization of manufacturing process. Most of the literature is focused on the investigation of process parameters to fabricate full dense parts showing good mechanical properties, or on the investigation of the effect of building orientation on the mechanical performance of components [11,12,16,21]. However, many authors recognized the importance of the quality of the raw material used in polymer powder bed fusion techniques [22,23,24,25]. Raw material quality greatly influences the powder-spreading process and, as a consequence, the quality of each deposited layer. In addition, Gibson et al. [26] presented evidence that the properties of the starting powder material strongly affected the mechanical properties of sintered parts when the process parameters were optimized.

A full understanding of the physical characteristics of powders in combination with their flow behavior is therefore fundamental to obtain high-quality components with desired and reproducible properties. Moreover, a preliminary evaluation of the relationship between the characteristics of polymer-based raw materials and the properties of resultant parts can help in making an informed choice of starting powders, whether they are commercial materials offered by different providers or new SLS powder systems developed for research purposes.

During the SLS process, the polymer powder is spread layer-by-layer by a blade or a roller that deposits a fixed amount of powder, theoretically without applying any external pressure. According to Wang et al. [27], the powder-spreading step is very important because it greatly influences the powder layer quality in term of density and homogeneity; moreover, the deposition method determines the possible forces exerted on the underlying solidified part. One of the main factors that must be considered when investigating the powder-spreading process is the geometry of the spreader. Generally, two types of recoater systems are used, a roller or a blade. Haeri et al. [28] reported that the use of a roller was more advisable with respect to that of a blade, because the former could produce a higher-quality powder layer. This was due to the fact that the roller showed a higher contact area, favoring a progressive rearrangement of particles during the spreading step. On the contrary, the blade exhibited a limited interaction with the particles, and a dragging effect occurred, leading to a rougher powder layer. Based on these considerations, Haeri [29] demonstrated that an optimization of the geometry of the blade profile in terms of width, height, and shape could significantly improve the powder bed quality.

Wang et al. [30] also compared systems with a roller and a blade; they found that the former allowed them to obtain a high-quality bed layer.

In addition to the use of a proper recoater, the quality of the deposited layer is strongly dependent on additional factors such as the dynamics of the powder. The understanding of how the powder interacts with the spreader and how the morphology of the powder pile changes during the recoater movement are critical issues that need to be investigated. However, the experimental evaluation of these dynamics is quite difficult. Some researchers [31,32] simulated the movement of powder particles during the spreading step by using a discrete element method (DEM). One of the properties that is generally used as an indicator for evaluating the powder behavior during spreading process is the powder flowability. Theoretically, any polymeric material that is available in powder form can be processed by selective laser sintering, if subjected by the laser beam to an incident power density suitable for sintering [1]. In practice, however, the properties of starting powders such as the flowability strongly influence the successful processing of materials. The powder flowability is defined as the ability of a material in the form of particles to flow in a desired manner when used in specific equipment [33]. Increase powder fluidity enables spreading of layers that are smoother, tighter, and more homogeneous.

Prescott and Barnum [33] underlined the difference between “powder flow properties” and “powder flowability”. The former refers to the existing interactions between the powder particles, which greatly influence the flow behavior. On the other hand, the flowability describes the flow behavior of a powder when used in specific equipment. This implies that the same material can show a different behavior as a function of different flow equipment used. For this reason, it is fundamental to characterize the powder flow properties under test conditions as similar as possible to those of the selected process.

Barletta et al. [34] distinguished three main methods to characterize the flowability of powders: static, dynamic, and defined consolidation.

Static methods such as the evaluation of Hausner ratio or the angle of repose determine the flow behavior of powder in static conditions. On the contrary, when the measurement setup (such as a powder rheometer) induces the powder flow, the characterization method is referred to as a dynamic method. Conclusively, the defined consolidation methods involve two main steps, including a first consolidation step followed by a shear step.

Ahmed et al. [35] provided evidence of the importance of evaluating the spreadability of a powdered system, in addition to its flowability. The two issues are inter-related, but different: in fact, the spreadability is a feature that allows the powder to be homogeneously distributed, forming a thin layer with a thickness up to a few particle diameters. A good spreadability implies that a continuous, defect-free, and uniform powder layer can be obtained. The proposed method used a Stanton cutter blade with different gaps to manually spread the powder layer. This latter was then analyzed by SEM to check its uniformity and the possible presence of defects. The main advantage in using this approach based on the preliminary analysis of the powder bed quality, was to verify the possible presence of small holes or agglomerates in specific spreading conditions.

Given the importance of powder flowability and the quality of the powder bed in PBF process, many authors investigated different factors that influence the homogeneity of the deposited layers, both by using experimental techniques [34,35,36,37,38] and modelling/simulations [28,39,40,41,42,43,44,45]. However, the evaluation of powder flow properties is quite complex, because it depends on many powder characteristics. It is worth noting that when polymer powders were mixed with different types of reinforcements such as fibers, graphene nanoplatelets, or carbon nanotubes, the evaluation of the system flow behavior was more complex [46,47,48]. In this paper, a definition of parameters that influence the powder flowability and their role in the SLS process is reported and discussed.

#### 2.1.1. Particle Size Distribution and Shape

The characterization of an SLS starting powder in terms of the determination of the particle size distribution (PSD) and the analysis of its shape constitutes a standard practice. Both these factors have shown a crucial role in the flowability of starting material: particles, which should have a geometry that allows a free flow when the recoater spreads them to form a thin layer; at the same time, these particles should present the proper size to be easily melted when the laser beam is applied.

The availability of polymer materials in powder form that show a particle size distribution and shape and that are suitable for the selective laser sintering process is still limited.

**Figure 3:**
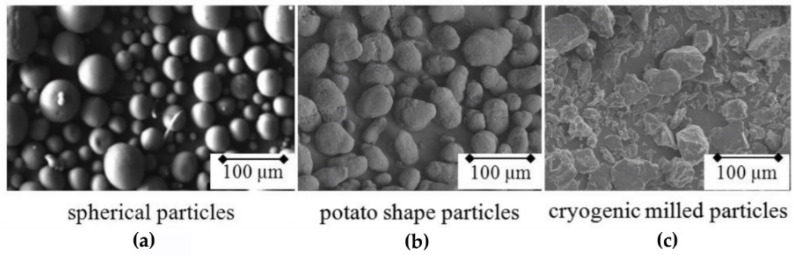
Particle shapes obtained by different production methods: (**a**) spherical, (**b**) potato-shaped, and (**c**) cryogenically milled particles. Reproduced with permission from [20].

The characterization of particle shape is generally performed by analysis of images that can be obtained using an optical microscope, scanning electron microscope, or X-ray microtomography. Different production processes can generate powder particles mainly showing three different shapes [20,24], as depicted in Figure 3. Spherical particles (Figure 3a) come from a coextrusion of a mixture of soluble and nonsoluble materials. The so-called “potato-shaped” particles (Figure 3b) are produced through a precipitation process; this configuration is the most common for PA12, the most widely used commercial material for SLS. Differently from the previous cited shapes, cryogenically milled particles (Figure 3c) show a random and very irregular geometry.

It is commonly accepted by the scientific community that spherical particles are the most suitable for the SLS process because they lead to maximizing the powder bed density and flowability [1].

Van den Eynde et al. [49] compared the quality of powder layers resulting from the deposition of three powders with different geometries: the very common commercial PA12 material showing a potato shape (the average size was 60 μm), polystyrene (PS) monodisperse spherical particles with different diameter, and cryogenically milled thermoplastic polyurethane (TPU) powders showing a very irregular particle size and shape. The experimental procedure involved the determination of various parameters such as the packing density (the ratio of tapped density and polymer material density), the packing ratio (which provides an index of powder flow), and quality of the deposited layer. The results showed that both PA12 and PS powders formed defect-free layers; on the contrary, the TPU layer was not continuous, showing many defects. Moreover, a comparison of the packing density showed that the spherical PS powder allowed the researchers to obtain higher values with respect to potato-shaped PA12 material.

Berretta et al. [50], who investigated the effects of particle morphology on the flowability of different polymer powders, evidenced that angular particles, as well as particles with irregularities and flakes on the surface, led to a final component with high porosity.

In order to maximize the powder bed density and obtain fully dense components, a suitable size distribution is required, in addition to a spherical shape [51].

Selective laser sintering generally involves the deposition of a powder layer with a thickness of about 100–150 μm [1]. Berretta et al. [52] reported that, to be sure that the powder melting occurred thanks to the direct interaction between the laser beam and the particles, rather than as a consequence of particle-to-particle heat conduction, the layer thickness should be with respect to the average size of the particles. However, the literature evidences some differences regarding the optimal particle size distribution reported by different researchers.

According to Wang et al. [53], the optimum particle size for powders to be processed by SLS was in the range between 45 μm and 90 μm, with a narrow size distribution. It is worth noting that particles with different size showed a different behavior during laser sintering: larger particles melted slower with respect to smaller ones. Differently, Chung et al. [54] affirmed that particles with a size in the range of 10 μm to 150 μm were preferred for SLS, because the PSD needed to be slightly smaller with respect to the layer thickness. Moreover, when using a powder with a wide size distribution, the optimization of process parameters required particular attention [51].

The optimal dimensional range was slightly different according to Schmid et al. [24]; their study reported that commercial powders to be processed by SLS needed to have a PSD in the range of 20 μm to 80 μm. However, the definition of a dimensional range was not sufficient to determine the processability of powders: the study compared two powders with similar volume distributions, but dissimilar number distributions (that is, a distribution in which the small particles had the same weight as the largest ones). Powder systems showing too high a fraction of small particles exhibited low flow properties.

Shi et al. [55], who investigated the effect of different powder materials’ properties on the quality of SLS parts, reported an inverse relationship between the particle size and the density of built components. In fact, particles larger than 100 μm led to a “step effect” and a low-density final component. On the other hand, when smaller particles were used, the flowability decreased, making the spreading step difficult. Therefore, the showed results evidenced that particles with a size ranging between 75 μm and 100 μm allowed the researchers to obtain high-precision and dense parts.

#### 2.1.2. Interparticle Forces

The selective laser sintering process involves the use of a blade or a roller that homogeneously spreads the starting powders. The formation of the layer is mainly influenced by the tendency of particles to rearrange and pack together to form a configuration that is as uniform and as dense as possible using their own weight as the main driving force. Gravity is the main force that can hinder the particle–particle cohesive forces that are possibly present in SLS systems [56]. However, according to Tayeb et al. [57], the role of these cohesive forces did not significantly influence the packing ability of the powders.

Therefore, the ability of a particle to flow is strongly dependent on the relative contribution of interparticle forces and particle weight, respectively.

Ruggi et al. [34] proposed an experimental procedure to firstly evaluate the interparticle forces from the flow properties, and then to determine the Bond number, which is the ratio between the interparticle forces for a nonconsolidated material and the particle weight. The results revealed that a Bond number lower that 100 was necessary to obtain a good-quality powder bed after the recoater passage.

Another experimental approach involved the determination of the Hamaker constant, a force parameter used for the evaluation of interparticle interactions while taking into account the physical and chemical nature of the powder materials under investigation [58]. When considering the interaction between two spherical particles, the smaller the Hamaker constant is, the weaker the Van der Waals’ forces should be, allowing for the free flow of particles. Berretta et al. [52] compared the flow behavior of two commercial SLS grade powders, PA2200 and HP3 PEK. The former was constituted of spherical or slightly elongated particles, while the latter contained particles that were not round. The results showed that the PA-based powders had the lowest Hamaker constant and better flow behavior with respect to the HP3 PEK system.

The evaluation of interparticle forces involved during the SLS process is more complex when a powder bed formed by nanoparticles is considered; in fact, at the nanoscale level, the interactions between particles are dominated by cohesive forces [59]. These can cause the formation of agglomerates that reduce the packing density, and result in the formation of porosity in the final component [56].

#### 2.1.3. Temperature and Humidity

During the SLS process, the powder bed is heated at a fixed temperature, usually in a range between the crystallization and melting points, in order to reduce the development of stresses in the built samples. Therefore, the flow properties should be evaluated not only at ambient temperature, when the spreading of homogeneous layer is fundamental, but in effective process conditions as well.

Amado et al. [60] used a modified rotating drum covered with a transparent glass in the inner side to record the powder behavior. Since the system was equipped with a heated cylinder, the study was able to investigate the influence of the temperature on the flowability of two commercial semicrystalline powders: PA12 and random co-polypropylene were studied from room temperature to 110 °C and 60 °C. The results showed that at temperatures higher than the glass transition point, there was a reduction in the surface fractal (a parameter describing the powder rearrangement); this may have indicated an increasing flowability, in addition to a homogeneous powder-packing deposition.

The strong influence of temperature on the powder flowability was also confirmed by Van den Eynde et al. [49]; they built a set-up that reproduced the spreading step involved during the SLS process. This equipment allowed them to evaluate the flowability through the ratio between the deposited layer density and the tap density, namely the packing index ratio (PIR). The results showed a direct relationship between this index and the density of the final component. It is worth noting that at temperatures above that of the glass transition, an increase in PIR was observed.

Ruggi et al. [34] proposed an alternative method to evaluate the influence of the temperature on the flow properties of powders. This defined consolidation method (see Section 2.4) used a high-temperature annular shear cell to evaluate the flow properties of a powder at temperatures close to those involved during the SLS process, in the range of room temperature to 180 °C. The results showed that at a temperature of 100 °C, the flow properties of the three investigated PA-based materials were optimized; this was in agreement with what was reported by Amado et al. [60], who evidenced an increasing flowability when temperatures higher than that of the glass transition were used. This was mainly due to a low humidity that limited the development of adhesion forces between the particles. When increasing the temperature to value very close to the melting point, a significant deterioration of the flow properties was observed. In this operating condition, the formation of capillary bridges and the plasticization of the contacts among particles may have involved an increment of interparticle forces, which led to a worsening of the flowability [61,62].

Berretta et al. [50] underlined the necessity of studying the effects of humidity on the SLS process, because it is responsible for electrostatically charging the particles. When the relative humidity is too high, particles generally charge negatively; on the other hand, too low a humidity tends to positively charge coarse particle and negatively charge fine particles. Optimal humidity conditions therefore need to be investigated.

### 2.2. Laser Motion and Irradiation (10^2^ μs)

#### 2.2.1. Optical Properties of Polymeric Powders

The interaction between the laser beam and the polymer powder bed is the starting point of the laser sintering process. In fact, the laser selectively delivers to the surface of the powder bed a huge amount of energy in a very short time (a fraction of a second) to melt the polymer grains [63]. The beam–matter interaction strongly depends on the laser wavelength, the chemical composition of the polymeric powders, and the particle size distribution. All these aspects define the material optical properties.

LS machines typically use CO_2_ lasers, which have a wavelength of 10.6 μm, because polymers show high absorption rates at long wavelengths. The midinfrared CO_2_ source excites the resonant vibrational modes (e.g., elongation or bending oscillations) of different segments of the polymer macromolecule, which are transformed into heat energy. Therefore, polymer powders can be considered as semitransparent materials due their chemical nature and granular state [64,65]. In fact, the supplied laser intensity is absorbed, transmitted, and diffused with multiple scattering inside the granular media, which has a size close to the laser wavelength [66]. The absorption characteristic of the polymeric powders at the specific laser wavelength adopted (known as absorptance), as well as the attenuation of the laser energy in the depth direction of the powder bed (known as the attenuation coefficient), are highly relevant in a successful sintering process. In fact, these properties affect the conversion of the optical energy delivered by the laser into heat energy, the penetration depth of the beam, and thus the dimensions of the melted zones [11,15,67].

Infrared spectroscopy (IR), such as Fourier-transform infrared spectroscopy (FTIR), was employed by different authors to assess polymer absorption characteristics [68,69,70,71]. These techniques provided valuable qualitative information on the absorption or reflection spectra of materials. However, the main disadvantage was the difficulty of quantitative analyses. In fact, IR experiments highly differ from SLS processing conditions (i.e., nature of the infrared source, sample preparation methods, temperature), and cannot describe the complex interaction between the laser beam and the powder material.

Experimental methods specifically designed for measuring laser absorptance, along with modeling efforts, are required to provide more insight on the beam–powder interaction. These methods can be divided into two groups [72]:

- The calorimetry method, which directly measures the absorptance from the temperature variations developed across a thin sample irradiated on the top surface;
- The radiometric method, which indirectly determines absorptance through the analysis of optical radiative properties such as reflectance and emissivity.

For opaque materials such as polymers, reflectance-based techniques provide an effective way to determine the material absorptance behavior.

Tolochko et al. [73] performed first measurements of the spectral reflectance and absorptance of laser sintering materials using an integration sphere (also known as an Ulbricht sphere) laser reflectometer. The instrument was equipped with both a pulsed Nd-YAG laser and a continuous-wave CO_2_ laser in order to study the optical properties of the powders at wavelengths of 1.06 μm and 10.6 μm, respectively. Different polymer powders, such as polymethylacrylate (PMMA), polytetrafluoroethylene (PTFE) and an epoxypolyether-based polymer, were analyzed. The results showed that the optical properties of all polymers strongly depended on the wavelength, with high absorptance values (between 0.73 and 0.94) at 10.6 μm, and very high reflectance (more than 0.90) at a wavelength of 1.06 μm. This behavior was explained by the different absorption mechanism of the polymers in different spectral regions. Moreover, the study demonstrated that a material displayed a much higher absorptance in its powder form compared to its bulk form. In fact, multiple reflections occurred in the voids between powder particles. These events resulted in a higher amount of radiation–particle interaction, and thus additional absorption, compared to dense materials.

**Figure 4:**
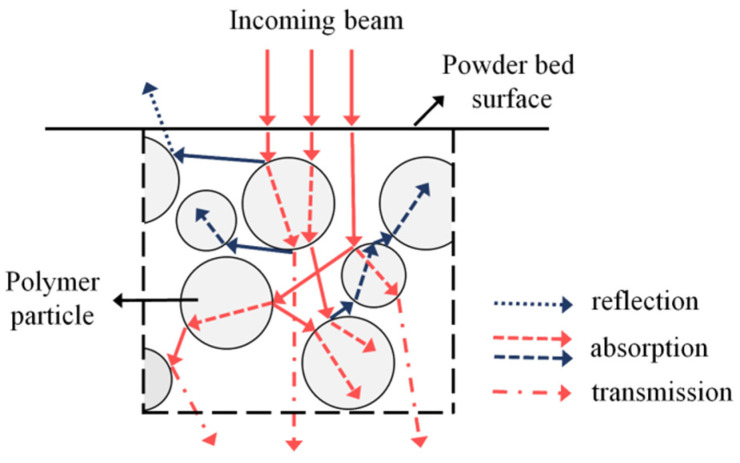
Graphical representation of the interaction of the laser beam with the powder bed, with multiple reflection/absorption events. Reproduced with permission from [75].

Laumer et al. [74] specifically designed an experimental set-up to investigate the optical material properties of polymer powders for SLS. The set-up was based on a double integration sphere that allowed the researchers to simultaneously measure the diffuse and direct reflectance, as well as the transmittance, of polymer powders for a CO_2_ laser source. The results showed that polyamide 12 (PA12) powders reflected only 5–6% of the incoming laser energy at the bed surface, yielding an absorption coefficient of 0.94. In contrast, the reflectance of polyethylene (PE) powder was significantly higher (30%). A detailed explanation model that took into account the optical properties of the bulk polymer and the multiple reflections within the powder bed was proposed to explain this difference (Figure 4).

The laser interacts with the powders in three ways: (1) reflection, (2) absorption, and (3) transmission. The net effect of these components is complex, as only part of the incident beam is absorbed at the outer surface. The other rays are repeatedly reflected due to the particle character of the powder bed. The reflected rays either propagate freely towards the surface or interact with other powder particles in their way. The portion of radiation that is absorbed and transmitted through a single particle depends on the absorption behavior of the polymer material itself. The absorption is determined by the distinct vibration oscillation of the polymer molecules. PA12 shows a strong absorptance at 10.6 μm due to the stretching vibration between the OC–C groups, as highlighted by IR measurements. In contrast, a weak rocking vibration of the methylene groups is responsible for the low absorptance of polyethylene at the same wavelength. Therefore, the different macromolecular structures of polyamide and polyethylene lead to different absorption/reflection behaviors of the powders.

Using this measurement approach, Laumer et al. [76] developed and qualified a PE powder for laser sintering by adding 1 wt. % of graphite. The graphite particles strongly absorbed the laser radiation, thus decreasing the reflectance of the PE powder bed from 30% to 10%. Multilayer parts with good interlayer bonding were produced with laser energy densities comparable to PA12, which is not possible without the addition of graphite.

**Figure 5:**
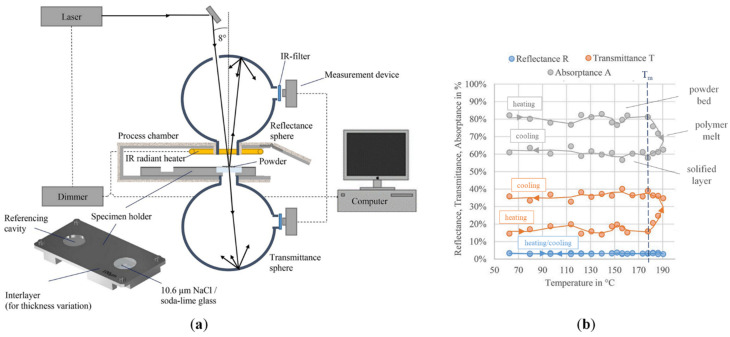
(**a**) Scheme of the integration-sphere measurement setup with a heated process chamber; (**b**) temperature-dependent optical properties of PA12 powder during CO_2_ laser exposure (layer thickness = 150 μm, laser wavelength = 10.6 μm). The heating and cooling cycle are outlined by arrows in all recorded curves. The dashed line at 182 °C corresponds to the PA12 powder’s melting temperature. Reproduced with permission from [77]; adaptation in (**b**).

In another work, Heinl et al. [77] investigated the influence of the temperature on the interaction between CO_2_ laser radiation and polyamide 12 powders. The integration sphere set-up, which was described in a previous work by the same research group [74], was updated with a heated process chamber to measure the optical properties of the powders in a temperature range relevant for SLS (60–190 °C) (Figure 5a). The results showed that the transmittance and the absorptance significantly changed depending on temperature due to the melting of the powder bed, while the reflectance remained almost constant (4%) (Figure 5b).

This behavior proves that the phase transitions of the polymer were responsible for these variations. In fact, the occurrence of melting led to fewer reflections between particles and lower scattering of the radiation on polyamide crystallites. Consequently, the transmittance increased from the initial value (16%) up to 35–40% after the melting point. During cooling, the melt solidified, and the orientation of the grown crystals produced an almost constant transmittance. Accordingly, the absorption was higher for the powder bed (80%) compared to the molten and solidified layer (61%) (Figure 5b). Furthermore, the authors studied the interaction of a short-wave radiation with the powders in order to provide insights on the development of an improved in situ optical monitoring system for AM technologies [77].

Schuffenhauer et al. [78] systematically investigated the scattering processes that occurred during SLS part fabrication between the incident CO_2_ laser beam and the polymeric powder bed. For this purpose, the double integration sphere set-up described by Laumer et al. [74] was adapted to collect both the unscattered and diffuse transmissions through polyamide 12 powders or thin films. Instead of sodium chloride, a barium fluoride (BaF_2_) infrared window was used, due to its higher transmittance and stability against humidity. The results showed that the transmitted power in a powder bed mainly consisted of diffuse radiation due to the multiple scattering between particle–environment interfaces. As a result, the penetration depth of the laser radiation was lowered, and the absorption occurred near the bed surface to the greatest extent. An increase in the absorptance as a function of layer thickness (from 60% at 150 μm to 90% at 300 μm) was also reported, in accordance with previous results [77]. Moreover, the scattering mechanism inside thin films that are were with different cooling rate was also analyzed. It was proved that the different sizes of spherulites, and thus the number of crystalline/amorphous interfaces between slowly cooled and air-quenched samples, strongly influenced the optical material properties. Higher transmittance was measured in films slowly cooled at 20 °C/min, approximately the average cooling rate reported for SLS of polyamide 12 parts [10].

The influence of multiple reflection and scattering, illustrated in Figure 4, on the absorption behavior of powdered material was validated for a metallic powder bed by Kruth et al. [63] using a 2D ray-tracing simulation model. The model allowed an accurate calculation of the laser beam penetration and the absorption profile along the powder bed depth. In fact, the path of most rays (representing the photons emitted by the laser) into the powder bed could be simulated by considering the reflection and refraction at the particle–atmosphere interface and the absorption inside a single particle. When a photon hit a powder particle, the amount of reflected, absorbed, and transmitted energy was calculated, and two new rays (reflected and refracted) were cast. These rays could propagate into the pores of the powder bed or hit another particle, causing new absorption, reflection, and refraction events.

Therefore, unlike in continuum-based models, the relevant transparency of granular materials was taken into account. These results proved that the laser–powder interaction in SLS involved complex phenomena that occurred not only at the outer surface, but also through the volume of the powder bed (Figure 4) [15,65].

**Figure 6:**
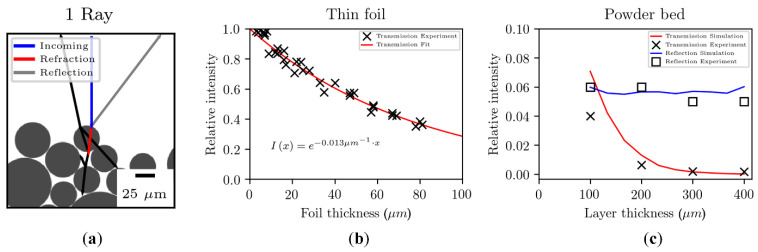
(**a**) Ray-tracing model describing the propagation of a photon through the powder bed; (**b**) relative transmission in PA12 thin foils as function of thickness; (**c**) comparison between the simulation results and experimental data for the relative transmission and reflection of a PA12 powder bed. Adapted from [79].

A similar model was developed by Osmanlic et al. [79] to examine the absorption characteristic and the attenuation properties of a thermoplastic polymer powder for SLS. The model traced the path of laser-emitted rays within a powder bed by analyzing the reflection and refraction at the particle–atmosphere interface and the absorption according to the Beer–Lambert attenuation law:

I = I_0_·e^−αx^ (1)

where I_0_ is the initial beam intensity (or heat flux), α is the attenuation coefficient for a certain wavelength, and x is the radiation path. The attenuation coefficient of PA12 was determined through infrared spectroscopy experiments performed on thin foils with different thicknesses. The relative transmission at a wavelength of 10.6 μm (corresponding to a wave number of 943 cm^−1^) was measured on 33 specimens, and an attenuation coefficient of 0.013 μm^−1^ was obtained (Figure 6b). The attenuation coefficient is the inverse of the optical penetration depth (OPD), which is defined as the depth where the transmitted intensity of the laser beam decreases at 1/e (37%) of the intensity of the incident radiation at the sample surface [66]. The model is graphically depicted in Figure 6a for a single ray to highlight the complex interaction between a photon and the powder bed. A good agreement between the simulation and previous experimental results [74] for reflectance and transmittance as a function of powder layer thickness was found (Figure 6c). Furthermore, the model suggested that the effective penetration depth in a powder bed could be lower compared to the bulk polymer because it was highly affected by the porous nature of the powder medium (i.e., relative powder bed density) and the multiple reflections through the powder layers.

**Figure 7:**
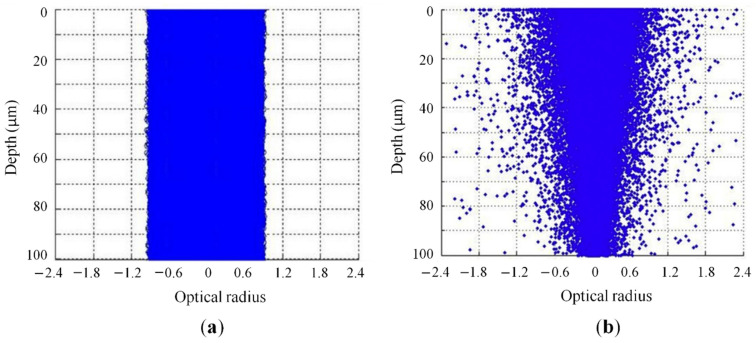
Simulation results of photon propagation inside a polymeric powder bed: (**a**) radiative model without scattering (the laser was considered a volumetric heat source, as proposed by Defauchy [80]); (**b**) Monte Carlo method with scattering. Reproduced with permission from [81].

The role of scattering on the interaction between laser and polymeric powders was further highlighted by Xin et al. [65]. The authors developed a ray-tracing model that was modified using the Monte Carlo method and the Mie scattering theory to simulate the radiative heat transfer due to localized heating of a granular media (i.e., metallic or polymeric powder bed) by a focused laser beam. The study accurately described the photon scattering and its influence on the radiative energy distribution within a polymeric powder bed. Without considering the scattering phenomena, all photons propagated longitudinally along the laser beam area, as depicted in Figure 7a. However, during the SLS process, photons randomly changed their propagation direction due to multiple reflections and scattering inside the powder bed. This led to two physical consequences that significantly affected the powder melting: the distribution area of photons was larger with respect to the laser beam diameter (Figure 7a); most of the absorbed laser intensity is concentrated close to the surface (Figure 7b), and the 2D spatial distribution of photons became gradually smaller with increasing depth. Therefore, the melted zone was larger at the surface compared with the bottom part of a layer.

Experimental studies of the laser penetration depth in a polymeric powder bed were firstly performed by Fan et al. [82] on the commercial TrueForm^TM^ (TF) powder (acrylic–styrene copolymer). For this purpose, a device was specifically designed to measure the transmitted CO_2_ laser energy through powder layers with variable thicknesses. A power meter, supported by a 95% transparent KCl window, was used to record the transmitted intensity. Powder layers with various thickness were obtained using different thin foils placed in the lateral part of the device. It was found that almost all the laser energy was dissipated within a thickness of 150 μm, which is around the layer thickness adopted in polymer SLS processes. The multiple reflections in the powder bed not only increased the optical pathways of the laser radiation, but also the number of rays reflected towards the surface. Therefore, more energy was absorbed at the bed surface. As a result, the transmitted laser intensity was reduced, and the peak temperature during sintering increased. These net effects are highly relevant if powders with fine particle distributions and high packing density are processed; this is due to a high number of available particle–atmosphere interfaces. In fact, among the three had investigated by Fan (2007), TF powders with a particle size lower than 45 μm have the lowest transmittance due to their lower sizes and higher packing fraction.

This phenomenon was also observed by Ho et al. [83] on polycarbonate (PC) powders. The authors measured the temperature of a point on the powder bed surface with respect to the time elapsed from laser irradiation using a noncontact infrared sensor. It was found that the peak temperature increased with decreasing PC particle size because the higher packing density of the powder bed led to additional energy absorbed near the surface.

These results were in good agreement with several experimental and modeling studies on polyamide 12 powders [74,77,78,79]. However, all the cited papers did not combine the experimental investigation of material optical properties with heat transfer analysis and evaluation of the dimensions of the melted zone after laser exposure.

**Figure 8:**
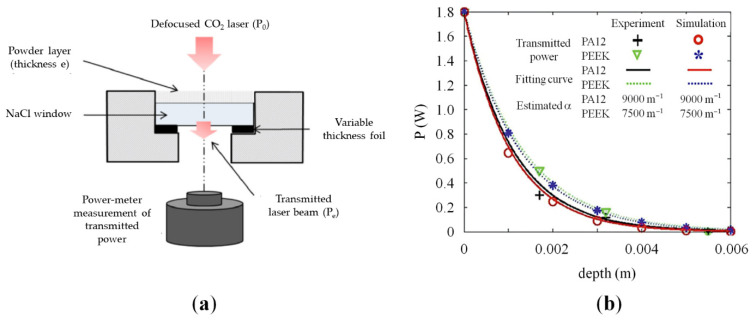
(**a**) Scheme of the experimental device used by Peyre [67] to measure the transmitted laser power through a power bed layer with variable thickness; (**b**) comparison between experimental results from Peyre [67] and DEM results from Xin [81] of the laser power transmission within PA12 and PEEK powder bed obtained using an incident power of 1.8 W. The symbols correspond to the measured and simulated values, and the continuous and dashed lines correspond to the Beer–Lambert law fitting that allowed the evaluation of the attenuation coefficient. Reproduced with permission from [67,81].

In a pioneer work by Peyre et al. [67], the attenuation properties of PA12 and PEEK powders were experimentally evaluated and coupled with a heat-transfer finite element (FE) model to simulate single fusion lines and layer deposition during the polymer SLS process. An experimental set-up, schematically sketched in Figure 8a, and similar to the device designed by Fan et al. [82] was employed to evaluate the laser/powder bed transmission for both powders. The results (Figure 8b) showed that the transmittances attenuated rapidly along the powder bed depth and became less than 10% of the initial intensity below 200 μm for PA12.

The experimental results can be described using the Beer–Lambert equation (Equation (1)). A higher attenuation coefficient was obtained for PA12 (0.009 μm^−1^) compared to PEEK (0.0075 μm^−1^), leading to an effective penetration depth of 110 μm and 130 μm, respectively. It was also found that estimating a correct value for the attenuation properties of the powders was important to accurately determine the melt pool size and thermal cycles during laser irradiation [67].

These experimental results were used by Xin et al. [81] to validate a multiphysical model, coupling radiative transfer, heat conduction, and sintering, of a polymer powder bed melting process. The simulation was based on a ray-tracing algorithm modified with a Monte Carlo method and the discrete element method (DEM) to describe the laser–powder bed interaction and the heat diffusion, respectively. The predicted values of the transmitted laser power and the attenuation coefficient yielded very good agreement with the experimental results (Figure 8b). Moreover, the study outlined that scattering defined the laser energy distribution inside the powder bed, and its correct estimation was extremely important to provide a realistic prediction of the width, depth, and temperature fields of the melted zones. Particularly, the estimation of the thermal history was useful to predict the evolution of the microstructure of the sintered layers, and thus the shape and final properties of the parts. These observations confirmed the strong relationship between the different physical phenomena involved in laser sintering [81].

#### 2.2.2. Effect of Filler or Absorbing Additives

Fillers are frequently used to improve the properties of laser-sintered polymers. Tolochko et al. [73] firstly reported the use of additives to increase the absorption efficiency of materials. Ho et al. [83] investigated the temperature variations within the powder bed during an SLS process in which additives that are commonly employed in the plastic industry such as graphite, quartz, silica, and talc were used. Graphite was observed to grant the highest temperature during laser exposure, as well as a slower cooldown compared to the other additives. Moreover, the study revealed that the addition of 2 wt % of graphite particles to polycarbonate powders significantly increased the surface temperature of the powder bed due to its higher absorptance at the CO_2_ laser wavelength.

This finding was confirmed by Laumer et al. [76], who studied the effect of graphite on the optical properties of PE powders using a double Ulbricht sphere, already described in the previous section (Figure 4). The results clearly demonstrated that the absorptance of PE with 1 wt. % graphite was increased up to 90% compared to 70% of neat PE powders. In fact, graphite particles almost fully absorbed the impinging radiation, leading to smaller penetration depth and reflectance of the powder bed. Therefore, graphite was suitable for improving the absorption characteristic of high-reflectance polymeric powders, and reduced the laser energy needed for PE sintering.

Wang et al. [70] analyzed the influence of graphite platelets on the absorptance of polyether ether ketone (PEEK) powders using FTIR analysis at a wave number of 943 cm^−1^ (equal to the wavelength of the radiation emitted from a CO_2_ laser source). The study showed that the absorptance of neat PEEK gradually increased by adding graphite to the neat PEEK powders. However, the flowability of the blended powders was gradually reduced by graphite platelets, leading to a higher number and size of pores in the sintered composites.

Other carbon fillers, such as carbon black and carbon fibers, have been used as radiation-absorbing additives to modify the optical properties of polymeric powders for SLS. Carbon black (CB) has proved to be effective in improving the absorptance of polyamide 12 [71,84] and polyether ketone powders [85]. Xi et al. [71] measured the reflectivity spectra of neat PA12, SiO_2_-coated PA12, and CB-coated PA12 powders through diffuse-reflectance infrared Fourier-transform spectroscopy (DRIFTS). The composite powders were prepared by dry-coating using a planetary ball mill. It was found that the reflectance at a wave number of 943 cm^−1^ largely decreased with an increasing amount of carbon black, from 20% of the neat powder to 11 and 7% when 1 wt. % and 2 wt. % of CB were added, respectively. On the contrary, SiO_2_ did not modify the optical properties of the base polymer. As a result, CB-coated powders absorbed the laser energy more efficiently compared to SiO_2_-coated ones. This led to higher melt pool temperatures and to an improvement of both the microstructure and tensile properties of the sintered parts. Fan et al. [82] and Lanzl et al. [86] also found that a glass microsphere and glass fibers had little effect on the reflectance of TrueForm^TM^ and polyamide 12 powder, respectively.

However, different authors [70,71,76,84,87] suggested that the addition of absorbing fillers such as graphite, carbon black, or carbon fibers reduced the optical penetration depth of the laser, because the radiation was mainly absorbed near the surface, and therefore did not propagate within the powder bed. Tian et al. [87] investigated the attenuation of laser energy with depth for different polyamide composites powders. It was found that the attenuation coefficient of PA12 reinforced with 40 wt. % carbon fibers was slightly higher than that of neat PA12. On the contrary, the value was much smaller when non-radiation-absorbing additives, such as NaCl, were added to the base polymer. This effect must be considered in the selection of the process parameters (i.e., layer thickness, laser energy input), otherwise the process could result in weak interlayer bonding. Furthermore, different research groups outlined that not only the optical properties (i.e., absorption, attenuation coefficient), but also the flowability, thermal conductivity, zero-shear-rate viscosity, and crystallization kinetics should be considered when evaluating the effect of reinforcing fillers on the processability of polymers in SLS [11].

### 2.3. Thermal Diffusion (10^2^ ms)

#### 2.3.1. The “Melt Pool”

The thermal energy that is irradiated from the laser beam is then transferred into the powder bed, leading to melting of polymer powder and to the formation of the so-called “melt pool” in the scanned region. The heat transfer is governed by the thermophysical properties of the powders, and directly influences the thermal gradient and temperature history of the melt pool.

**Figure 9:**
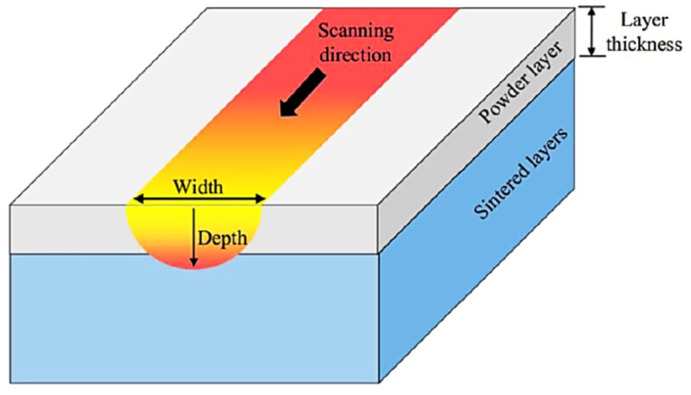
Schematic view of the melt pool during laser irradiation. Reproduced with permission from [75].

The melt pool is usually characterized in terms of dimensions, specifically depth and width, and temperature distribution. These quantities, which are graphically depicted in Figure 9, play a significant role in the polymer SLS process, and the understanding of the dynamics of the melt pool is essential to obtain a viable process.

The melting depth is crucial to ensure the consolidation between two consecutive layers. This is essential to produce specimens with good mechanical performances; in fact, an incomplete fusion between two successive layers does not lead to complete densification, thus causing the formation of voids, and consequently, of parts with poor mechanical properties. There are some cases in which porosity is required, such as filters and components for application in the biomedical field. The porosity can be tuned by decreasing the energy provided by the laser beam [88]; however, this method cannot precisely control the pore content and morphology. Therefore, other approaches have been proposed in recent years. One approach is the production of lattice structures during the creation of a CAD 3D model [89]. Another method, reported by Tian et al. [87], consisted of mixing powders of different natures; for example, polymeric and inorganic materials. The inorganic powders could be dissolved after the SLS process, leaving the desired porosity degree.

**Figure 10:**
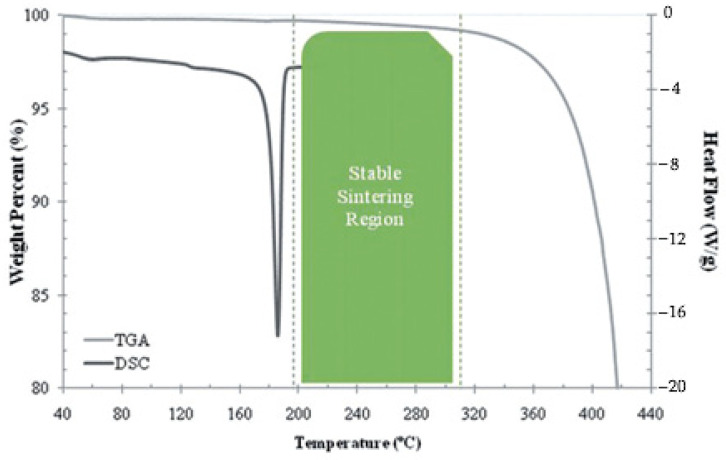
“Stable sintering region” for thermoplastic polymers. Adapted from [90].

Melting depth also determines the allowable thickness of the deposited layer, and it can thus be related to the process resolution. According to Shen et al. [75], the melting depth must be higher than the height of a single powder layer in order to achieve full melting of the material, but should be lower than twice the height of a single layer; otherwise, an excess remelting can occur, which might cause high thermal stresses and even degradation of the polymer. The melting width is related to the transversal influence of laser radiation—its value is connected to the hatching space; i.e., the distance between adjacent scanning lines, and its knowledge can be useful for efficient preprocess settings. Finally, the temperature distribution is crucial to the properties of the manufactured object. Vasquez [90] identified a range of temperature values within which the melt pool should remain. This range is called the “stable sintering region”, (SSR) and it is graphically shown for PA12 powder in Figure 10. The SSR is included between the end of the endothermic melting peak, which can be obtained using DSC, and the onset of degradation of the polymer, given by TGA analyses. Beyond the aspects related to degradation, Shen et al. [75] suggested an upper limit of around 300 °C in the studied case of CNT-coated PA12 powder; this upper limit was established in order to preserve the dimensional accuracy of the object, as the polymer particles near the borders of the layers risk being melted at temperatures higher than 300 °C.

According to Lupone et al. [91] the width of the SSR can be further narrowed around its average value. Experimental results obtained starting from two very different polymer-based powders, PA12 reinforced with carbon fibers and polypropylene respectively, showed that the best combination in term of part densification, dimensional accuracy, and mechanical properties could be obtained using energy density values within the middle part of the SSR.

#### 2.3.2. Main Process Parameters Involved in the Formation of the Melt Pool

The formation and evolution of the melt pool arise from the interaction between the laser irradiation and the powder bed; consequently, the properties of the melt pool strongly depend on the adopted process settings. The main parameters of SLS process are strictly related to the laser beam; these are the laser power, the laser scanning speed (the speed at which the laser beam moves on the powder bed surface), the hatch spacing (the distance between the center points of the laser beam profile in subsequent rasters within one layer), and the laser beam diameter.

Several studies have found it convenient to refer to the energy density *ED*, which was defined by Drexler [92] as:

(2) $$\mathrm{ED}=\frac{P_{L}}{h_{s}\times d_{l}\times v_{s}}\;(J/{\mathrm{mm}}^{3})$$

where P_L_, h_s_, d_l_, and v_s_ are the laser power, the hatch spacing, the laser diameter, and the scanning speed, respectively. This parameter quantifies the interaction between the laser beam and the powder bed through the hatch spacing and the laser diameter, as well as the amount of the involved energy through the laser power and the beam speed. In the work of Starr et al., an alternative parameter; that is, the volume energy density ED*_vol_*, was used to quantify the energy density input. The authors reported that this parameter can be useful when the layer thickness (z) might be changed [93]. ED*_vol_* was defined according to Equation (3):

(3) $${\mathrm{ED}}_{vol}=\frac{P_{L}}{h_{s}\times z\times v_{s}}\;(J/{\mathrm{mm}}^{3})$$

Starr also introduced the concept of the energy–melt ratio *EMR*, which was defined as the ratio between the volume energy density ED*_vol_* and the energy needed to cause the full melting of the unit volume of the powder layer *E_m_*. EMR can be calculated by knowing the values of the bed temperature and the properties of the powder, such as powder packing density, specific heat, melting temperature, and heat of fusion [93]. As underlined by Berretta, *EMR* is the most comprehensive factor that combines both processing parameters and powder properties [94].

Several studies focused on the evaluation of mechanical properties of SLS-processed specimens when varying the process parameters. Caulfield et al. [95] studied the influence of an energy density that was varied by changing the laser power at a fixed value of laser scan spacing on the physical and mechanical properties of PA12 specimens. The increase in energy density led to higher values of the part density, which could be explained by the fact that higher energy density values caused a better fusion of the powders, resulting in a more solid part being manufactured. This also led to an improvement in the tensile strength and Young’s modulus. Nevertheless, this improvement was confined until a certain temperature was reached, after which the mechanical properties began to decline. According to Vasquez, this phenomenon could be attributed to the onset of degradation of the polymer powder, which might also have led to the local evaporation of the material, causing the formation of bubbles, and resulting in part porosity [90]. Similar results were obtained in the work of Starr et al. [93], who studied the evolution of mechanical properties as a function of energy density, volume energy density, and energy–melt ratio. Both tensile strength and elastic modulus showed an increase with increasing energy density, achieved by varying in turn the laser power or the laser scanning speed, until plateau values were reached for both properties at high energy levels. Analogous trends were obtained in the study by Vasquez, who investigated the variation of mechanical properties of PA12 and TPU samples by varying the energy density, which was increased by decreasing the values of the scanning speed or by increasing the laser power; tensile strength, elastic modulus, and elongation at break increased linearly with energy density, independently from the way the values of ED were raised.

#### 2.3.3. Powder Properties Affecting the Melt Pool

In addition to the process parameters, the dynamics of the melt pool are strictly related to the thermophysical properties of the polymer powders, which directly influence the dimensions and the temperature of the melt pool.

The heat flux along the melt pool is mainly controlled by the heat capacity and the thermal conductivity of the polymer. These material characteristics depend on the physical phase of the polymer, as well as on the porosity and on the temperature [96]. In the study of the evolution of the melt pool, these quantities may be expressed either using models, or by deriving their trend using DSC measurements, or eventually by using their average values in the investigated temperature range; this will be discussed more in detail in the next paragraph. In addition, in order to have a positive outcome of the process, the polymeric powders must possess a wide super-cooling window. This term is defined as the temperature gap between the onset of melting and the onset of the crystallization event [94]. This range should be wide in order to ensure that the powder contained in the powder bed does not melt before exposure to the laser and does not crystallize before the end of the process, in order to avoid distortions of the printed objects. Furthermore, Vasquez et al. performed DSC measurements on SLS-grade PA12 and TPU powders, suggesting that a high heat of fusion and a narrow melt peak were beneficial in the formation of a well-defined melt pool, in order to prevent the melting of the powder close to the melt pool perimeter [97].

In addition, optical properties of the powder have an influence on the evolution of the melt pool. It is useful to note that the optical response of the powder bed to the laser irradiation should provide high levels of absorptance to achieve full melting. The studies by Tolochko et al. and Laumer et al. demonstrated the dependence of the optical properties, in particular the absorption, on the used wavelength, and that at a fixed wavelength (i.e., the same laser source) different materials had very different behaviors; furthermore, the optical absorptance of a polymer powder bed presented higher absorptance values compared to the same material, but in bulk state [73,74].

#### 2.3.4. Evaluation of the Melt Pool: Modeling and Experimental Approaches

As said previously, for a successful outcome of the process, it is crucial to gain a deep understanding of the mechanisms involved in the formation of the melt pool. For this reason, several studies have focused on the evolution of the melt pool and its properties, relating them both to the material properties and the process parameters. In order to evaluate these aspects, both numerical modeling and purely experimental approaches have been exploited. It should be pointed out that both methods showed some limitations in the effectiveness of the predicted results. Numerical modeling, in fact, still requires a deep understanding of the phenomena related to the polymer laser sintering process, which seemed to be the most complex and the least understood among those described in literature, as they involved deep interactions between heat, mass, and momentum transfer, together with chemical modifications of the materials and variation of mechanical and thermophysical properties. Conversely, purely experimental approaches were necessarily specific, and they might not have been feasible to describe all the aspects related to the phenomena of the process [98]. Several studies combined both the approaches, with the aim to validate the proposed models through the experimental results.

**Figure 11:**
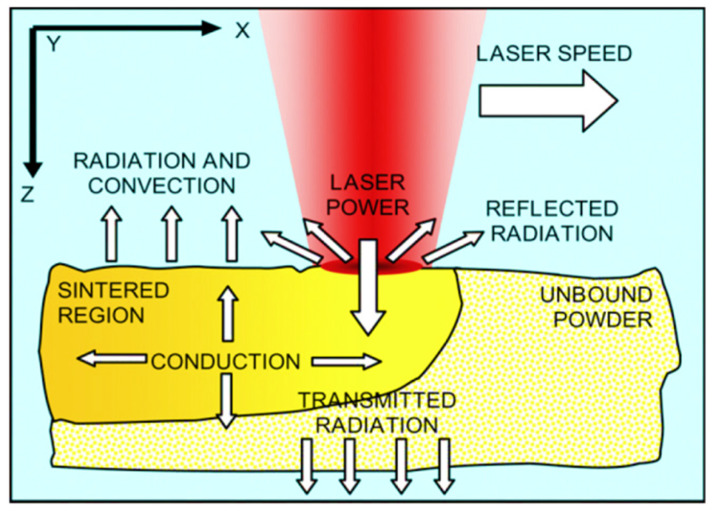
Schematic representation of energy-transfer phenomena occurring in SLS. Adapted from [98].

In most of the studies, modeling of the melt pool was based on a heat-transfer problem. The energetic phenomena that take place during the formation of the melt pool are schematically represented in Figure 11. The laser beam, which is the heat source, irradiates the powder bed; the energy is either transmitted, reflected, or absorbed, depending on the optical properties of the powder; heat transfer through conduction, controlled by the thermal properties of the polymer, takes place inside the melt pool, while radiation and convection phenomena occur at the interface between the powder bed and the chamber. Other heat sources that are normally present are the IR lamps and the heaters, which are needed to warm both the chamber and the powder bed.

In a real process, there might be small variations in the bed temperature due to several reasons related to limitations of the machine, such as irregular heating elements, conduction through the part walls, thermal convection channels that form in the N_2_ atmosphere above the part bed, or conduction through the part chamber walls [14]. Although these aspects could influence the evolution of the melt pool, it was difficult to implement them in numerical models of the thermal diffusion during polymer SLS.

**Figure 12:**
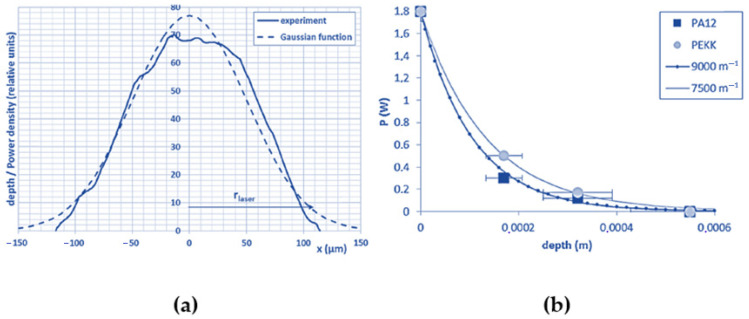
(**a**) The 2D beam profile distribution of the laser; (**b**) laser/power transmission measurements. Adapted from [67].

The laser source is normally modeled using a Gaussian function to describe the laser beam distribution along the plan perpendicular to the direction of the beam propagation; i.e., the *x*–*y* plane. Peyre et al. estimated the beam profile distribution, using a 2D profilometer to measure the deformation resulting from the laser-induced fusion on a dense polymer, and the resulting distribution, reported in Figure 12a, was shown to be near-Gaussian in shape [67]. They proposed the following equation to model the surface heat flux due to laser irradiation:

(4) $$Q_{0}=Kn\;\frac{P_{0}}{\left(\pi r_{laser}^{2}\right)}exp\left(-n\frac{\left(x^{2}+y^{2}\right)}{r_{laser}^{2}}\right),$$

where *n* and *K* are coefficients derived from the analytical solution of the model.

Regarding the transmission of the laser along the z-direction; i.e., the direction of the depth of the powder bed, it was modeled in most of the studies using the Beer–Lambert equation:

Q(z) = *Q*_0_ *exp*(−αz) (5)

where α is the extinction coefficient and *Q*_0_ the surface heat flux.

In the previously cited work, Peyre measured the transmission of a laser beam through a powder bed using different polymers, and the results, reported in Figure 12b, showed an exponential decrease in transmission along the powder bed depth, according to the Beer–Lambert equation [67]. The transmission of a laser beam through a powder bed was also modeled by Osmanlic et al., and their work also brought them to the conclusion that laser transmission could be modeled using the Beer–Lambert law [79].

It should be noted that common lasers used in the SLS process operated in pulsed regime, while the laser irradiation was commonly modeled as a numerical continuous heat deposit. Peyre demonstrated that this assumption was valid for speeds lower than a certain threshold speed, while above this value, the laser irradiation could not be considered a continuous heat deposit anymore [67].

The extinction coefficient is related to the optical behavior of the polymer powders, and it strongly affects the evolution of the melt pool. Tian et al. modeled the dynamics of the melt pool for PA12, PA12/CF, and PA12/NaCl powders; while the first two materials showed similar extinction coefficients, the PA12/NaCl powders showed a lower value of this magnitude, due to the transparency of NaCl to the CO_2_ laser. This led to a different evolution of the melt pool, as the PA12/NaCl powders exhibited a much higher depth of the melt pool, which was determined using the temperature-distribution profile [87]. Nevertheless, as PA12 is the most employed material in SLS, absorption data often refer to this material. PA12 showed values of absorbance higher than 0.95 [74]; for this reason, transmission and reflection phenomena are often neglected in the modeling of the melt pool.

Conduction phenomena are responsible for the heat diffusion through the powder bed, and they are related to the heat conductivity *k* and heat capacity *C_p_* of the polymer. Both these quantities can be described using models, or their values can be obtained using DSC measurements. Heat capacity was modeled in the work of Dong et al. as a function of porosity and the heat conductivity of the solid material, which in turn was a function of temperature [96]. Riedlbauer measured the values of *k* using DSC and interpolated these measurements as a function of temperature; this derived function was also used in the work of Shen to model the values of *k* with the temperature change [75,99]. Peyre used a time-dependent Heaviside step function to simulate a variation of thermal conductivity during the layer-deposition sequence [67]. Finally, in other works, heat conductivity was kept constant at a fixed value [18,67,90,98].

Regarding heat capacity, Dong modeled the *C_p_* of polycarbonate as a linear function of the temperature in both the solid and the melt phases [96]. As for *k,* Riedlbauer measured the values of *C_p_* using DSC and interpolated these measured values as a function of temperature; *C_p_* showed a peak at the transition between solid and molten phase, due to the latent heat of fusion [99]. Peyre and Shen used a Gaussian function to describe the behavior of *C_p_* with the temperature change, in which the peak was also due to the solid–melt transition [67,75]. Again, other works assumed *C_p_* to be constant during the process [18,67,90,98].

Modeling often locates convection and radiation phenomena at the powder bed surface. Dong modeled these terms with the following equation [96]:

(6) $$-k_{e}{\frac{\partial T}{\partial z}|}_{z=s}=h\left(T_{a}-T_{z=s}\right)+\varepsilon_{R}\sigma \left(T_{a}^{4}-T_{z=s}^{4}\right)$$

where *T*(*z* = *s*) is the temperature on the powder bed surface, *T_a_* is the preheating environment temperature, *h* is the convective heat transfer coefficient, *ε_R_* is the emissivity of the material, *σ* is the Stefan–Boltzmann constant, and *k_e_* is the effective thermal conductivity. The same contribution was considered also in the works of Peyre [67] and Mokrane [15]. Convection and radiation terms, however, were considered to be negligible in different works, due to the small exchange surface and low temperature, and also due to the fact that laser motion and thermal diffusion are phenomena that take place in the time of microseconds or milliseconds, so conduction was the most relevant phenomenon [18,67,75,98].

A boundary condition that was commonly considered in the various models was the initial temperature on the surface of the powder bed; furthermore, adiabatic conditions at the boundary of the powder bed were often assumed [18]. Finally, modeling could be done either considering the powder bed as a homogeneous material, or by considering it as a granular medium; nevertheless, it was not the purpose of this review to investigate in depth the different typologies of modeling techniques.

The majority of the works that involved modeling of the melt pool focused on the influence of the process parameters on the dimensions of the melt pool and on the temperature profile. These quantities were also measured using an experimental approach: melting depth and width were often measured, while processing by SLS, a single scan line whose dimensions were measured using several microscopy techniques (stereomicroscopy, optical microscopy, or electron microscopy); in addition, the temperature on the surface of the melt pool could be measured using infrared cameras.

In general, the dimensions of the melt pool and the values of the temperature increased with increasing energy density. More in detail, considering the parameters involved in the definition of ED, the temperature, melting depth, and width increased with increasing laser power and decreasing scanning speed, as the powder bed was subjected to a higher energy input. Dong modeled the dynamics of the melt pool during SLS of polycarbonate; the results of the model showed that the maximum temperature increased linearly with increasing laser power, while it decreased with increasing scanning speed [96]. Similar trends also were found in the works by Peyre, who performed both experimental and numerical analysis of SLS of PA12 and PEKK semicrystalline polymers [67]; Shen, who modeled and experimentally validated an SLS process of PA12 and PA12-composite powders [75]; Yuan, whose work focused on numerical and experimental study of SLS of TPU powders [100]; and Bierwisch, whose work aimed at the development of universal process diagrams for SLS using a numerical approach [18]. The dimensions of the melt showed an increase with increasing ED values as well, but this rise was less rapid if compared to the values of maximum temperature. Yuan suggested that both depth and width of the melt pool increased as a logarithm function of energy density [100]; these trends of melting depth and width were comparable to those obtained in other works [75,98,99]. Drummer et al. evaluated the impact of heating rates on the melting depth and width, producing single-scan lines with the same ED values but different heating rates; the values of heating rates involved in the process were extremely high, in the order of 10^7^ K/min [101,102]. The authors showed that melting width was increased at higher heating rates, causing a denser molten layer, while the melting depth was reduced by increasing heating rates, which resulted in a weaker connection between layers.

### 2.4. Polymer Viscous Flow and Particle Coalescence (10^1^ s)

#### 2.4.1. Binding Mechanisms in SLS

Although the term “sintering process” commonly refers to the formation of objects through consolidation of grains at high temperatures, but still lower than the melting point, the binding mechanisms occurring in selective laser sintering of polymers do not involve solid-state diffusion phenomenon. The latter is in fact a rather slow process, and it is not compatible with the high speeds related to the laser scan in the SLS process. Nevertheless, the terminology has been used in many works when referring to polymer coalescence, and it is now well accepted in the literature.

The consolidation of polymers during SLS involves mechanisms that are still believed to be amongst the least understood in the different classes of materials processed by powder bed fusion techniques, as pointed out by Kruth et al. [103]; nevertheless, in most cases, the mechanism of consolidation involves the melting of powders. It is possible to refer to partial or full melting in the case of semicrystalline polymers, while the mechanism of consolidation of amorphous polymers can be considered as a liquid-state sintering process, as these do not exhibit fusions. The classification of a certain process may also fit in more than a single category; Kruth et al. [104] reported the example of glass-filled polymers, the SLS process of which could be seen either as a liquid-phase sintering, with the polymer as the binder and the glass particles as the structural material; or a full melting process, in which the composite was considered a single structural material that was fully molten.

One technique that can be used to determine whether partial or full melting occurred in the SLS process is the evaluation of the crystalline structure of SLS-manufactured parts, using both microscopy and DSC analyses. Zarringhalam et al. [105] studied the microstructure of SLS PA12 samples that were also compared to the crystalline structures of the employed powders. The microscopy analysis showed the presence of particle cores surrounded by spherulites, as well as spherulites without cores. The presence of cores could be attributed to the incomplete melting of particles due to insufficient heat, while spherulites without cores were formed from particles that were small enough to fully melt. DSC analyses confirmed the hypothesis of a partial melting, due to different peaks related to the different crystalline structures of the unmolten cores and the melted and recrystallized regions. Similar result were obtained by Dadbakhsh et al. [106], who evaluated the melting behavior of SLS-processed PA12 parts; even in this case, DSC heating ramps registered two separate peaks related to the fusion of different crystalline structures related to the unmolten particles, and to the molten and subsequently crystallized regions. WAXS analyses better identified the crystalline structures of these two different phases, attributing a metastable α-phase to the powders, while molten and recrystallized regions featured the more stable γ-phase.

#### 2.4.2. Models of Viscous Sintering

**Figure 13:**
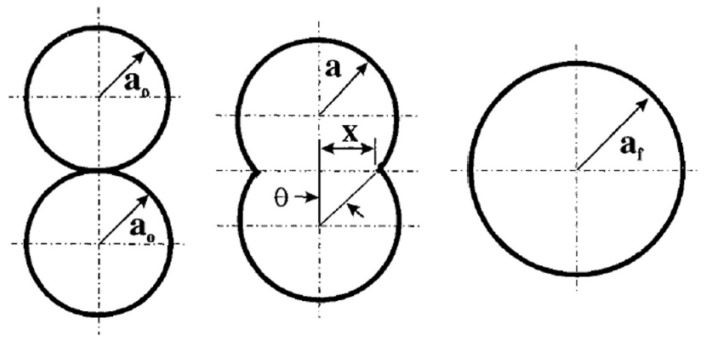
Schematic representation of particle coalescence according to Frenkel’s model. Reproduced with permission from [108].

In any case, whether partial or full melting occurs, it is acknowledged by now that consolidation phenomena in selective laser sintering of polymers are mainly due to viscous motion [107]. Several models of coalescence driven by viscous flow can be found in the literature; one of the first studies that predicted the coalescence behavior was proposed by Frenkel et al. [11]. According to this model, the rate of coalescence is driven by Equation (7):

(7) $${\left(\frac{x}{a_{0}}\right)}^{2}=\frac{3\sigma t}{2r\eta_{0}}$$

where *x* is the length of the growing neck between the two adjacent particles, *a*_0_ is the initial radius, *σ* is the surface tension, *η*_0_ is the zero-shear viscosity, and *t* is the time; a graphical explanation of the process and the involved quantities is shown in Figure 13. This model was found to be mainly valid in the first steps of the coalescence process, during which particles maintain a spherical shape; according to Frenkel’s model, coalescence can be considered to be completed when the radio $\frac{x}{a_{0}}\;$ is equal to 1.26 [108].

Molten polymers are known to feature a complex rheological behavior, as viscosity can significantly vary as a function of temperature, shear rate, and time (due to chemical modifications of the polymers’ structure), so further studies have reported models that better fit the rheological viscoelastic behavior or polymers. In a study by Sun et al. [109], the sintering process for a viscous powder bed was modeled by describing the powder bed as equal-size spherical particles aligned in a cubic way. This modeling choice could be explained due to the similar density between the powder bed and the cubic structure. In a study published by Pokluda et al. [110], Frenkel’s model was modified in order to simulate the coalescence of two spheres throughout the whole process, and not only for the initial stages, with the assumption of a constant shear rate. Bellehumeur et al. [111] combined both the upper and lower Maxwell models with Frenkel’s model in order to consider the viscoelastic nature of molten polymers; this model, together with experimental results, showed the importance of viscoelastic effects in the sintering process of polymer particles, as the process rates appeared to be slower for polymers with a higher elasticity content.

Nevertheless, the model proposed by Frenkel highlights two material properties that play a key role in the process of coalescence, which are the surface tension and the viscosity.

There are different methods for the measurement of surface tension of molten polymers, such as the Wihelmy method and the Punch method. The value of this property, which was in the order of 10^1^ mN/m, did not significantly vary as a function of temperature and polymer type [112]. The Wihelmy method, which was studied by Sauer et al. [113], could provide measurements of surface tension by soaking a fiber into the polymer melt; from the measured wetting force, the fiber diameter and the contact angle, it was possible to determine the values of surface tension. On the other hand, the Punch method [102] could determine the values of surface tension employing a metal punch, on which the molten polymer was placed. The melt wetted the surface of the punch to form a drop; the surface tension of the melt could then be calculated from the drop’s geometry using the Young–Laplace equation. Another approach developed by Sugden allowed a correlation of the surface tension with the molecular structure, used to define the value of an empiric parameter named Parachor. This quantity correlated the chemical structure of the polymer with free energy and molecular volume, according to the following equation:

(8) $$P=\frac{\gamma^{1/4}\times MW}{d}$$

where *P* is the Parachor, *γ* is the surface energy, *MW* is molecular weight of the repeat unit, and *d* is the molar density of the repeat unit of the polymer [11].

As surface tension lies between a narrow range of values, it is viscosity that mainly influences the sintering process. In fact, the viscosity gives an indication of the fluidity of the polymer melt; high values of viscosity lead to a higher difficulty to flow, resulting in slower coalescence rates. Polymers are viscoelastic fluids, meaning that viscosity is a function of both temperature and deformation rate, in contrast to Newtonian fluids, the viscosity of which changes only with temperature. Viscosity of melt polymers normally shows two different trends depending on the shear rate values: at low shear rates, melt polymers tend to behave like Newtonian fluids, as viscosity tends to present stable values; i.e., the so-called Newtonian plateau. On the contrary, at higher shear rates, the viscosity sharply decreases with increasing frequency, in what is the shear-thinning region of the curve.

In the SLS process, no deformation rate is applied to the polymer melt, as laser selectively fuses the polymer powder, which then coalesces under the only force of gravity. For this reason, the most studied rheological quantity in the field of SLS is the zero-shear viscosity. An appropriate value of this parameter is often seen as a prerequisite to obtain full consolidation of the part, and for this reason, rheological measurements are often performed prior to the SLS process in order to understand whether a material can or cannot be employed in the process. Furthermore, a prediction of the rheological behavior of the melt during the SLS process is not trivial, as the technology itself involves rapid changes in temperature, which deeply influences the values of viscosity. In this regard, an interesting approach was proposed in the work by Yan et al. [114], who studied the rheological behavior and sintering kinetics of CF/PEEK composites during SLS. In this work, a numerical model was used to predict the temperature distribution along the melt pool, to eventually describe the melting pool as a function of zero-shear viscosity. The study considered 10^5^ Pa·s as the highest acceptable value of viscosity in order to promote coalescence. However, this value was much higher than the limit of 60 Pa·s obtained in the theoretical model proposed by Shi et al. [55]; moreover, it was also much higher than SLS-grade PA12 viscosities. Nevertheless, it was chosen as a reference because it is generally considered the upper limit for injection molding. The results of the study highlighted the importance of viscosity in the process of consolidation, as the materials with high values of viscosity resulted in a less dense microstructure and poor mechanical properties, compared to the materials with low viscosity.

The viscosity of melt polymers can be measured using different methods. Rotational rheometry can provide reliable values of viscosity at low values of shear rate, which corresponds to the rotational speed of the plates of the rheometer; this technique is thus the most used in order to obtain values of zero-shear viscosity. On the other hand, capillary rheometry is used when higher deformation rates are involved. Another measurement technique that gives an indication of the fluidity of the melt polymer is the melt flow index (MFI): it provides the amount of mass flown in a fixed time range through a capillary of certain length and diameter. Although there is not a direct correlation between the MFI and the viscosity values, MFI measurements were used to provide indices of fluidity in the studies of Kim [115] and Berretta [116].

PA12 is by far the most widely employed polymer in SLS, so several studies can be found in the literature concerning its rheological behavior; in addition, this material is often considered as a benchmark to verify the feasibility of other materials to be processed by SLS [97]. The study of Haworth et al. found values of zero-shear viscosity of 390 Pa·s for virgin PA12, while used PA12 (meaning powder that had been through a process cycle, and therefore subjected to an LS bed temperature of around 170 °C for a period of up to 3–4 h) presented values of 5095 Pa·s, one order of magnitude higher [117]. An increase in viscosity of aged PA powder is a well-reported phenomenon, and it is believed to occur due to postcondensation at solid state, leading to an increase in average molecular weight, and consequently in viscosity; the increase in viscosity in turn causes a higher sintering time. Zarringhalam [105] demonstrated the increase in molecular weight in used PA12 powder using gel permeation chromatography (GPC); the weight changed from 70,000 to 170,000 g/mol, and resulted in a higher melting point of the SLS-made part. Similar results were obtained by Dadbakhsh [106]: GPC confirmed the increase in molecular weight in used powders, while measurements of the zero-shear viscosity indicated an increase in η_0_ with increasing time of exposure at high temperatures. This increment was more significant at higher temperature, suggesting that postcondensation phenomena were favored in these conditions. Postcondensation is a phenomenon that occurs not only in PA12, but in most polymers. Verbelen et al. [112] studied the trend of η_0_ with time in different polyamides powders (PA12, PA11, PA6), and all of these materials, excluding one type of PA12, showed an increase in η_0_ with time that was most significant with high temperatures. Postcondensation of PEEK and its composites was examined in a study by Yan, who demonstrated a slight increment in η_0_ with increasing time [114].

Another aspect that was examined in several studies was the influence of the presence of fillers on the rheological behavior of the starting powders; this issue must to be investigated to verify the feasibility of the production of composites using SLS. It is known that the presence of the fillers causes an increase in viscosity; this phenomenon can be explained by the interaction between the polymer and the filler, as the latter hinders the movement of the polymer chains, thus decreasing their mobility and increasing the viscosity. Kim et al. [115] studied the sintering characteristics of nylon 6 and clay-reinforced nylon 6; rheological measurements showed that the addition of clay lowered values of the melt flow index (MFI), hence producing higher values of viscosity. Furthermore, sintering experiments indicated that the final density of clay-reinforced nylon 6 was lower than that of the sintered pure polymer, due to the higher viscosity of the composite powders. Similar results were obtained in the work of Yan [114], who showed that carbon-fiber-reinforced PEEK exhibited higher values of viscosity than pure PEEK, indicating that higher temperatures were needed for the composite powders to reach the same flowability of pure PEEK. Finally, Bai et al. [118] studied the influence of carbon nanotubes on the rheology of PA12 powders. As previously discussed, the addition of the fillers caused a marked increase in the values of viscosity. However, an unexpected result was the increase in viscosity with increasing temperature, which was explained through the formation of entanglements between chains at higher temperatures that were not present at lower temperatures due to the presence of voids and/or unmolten particles.

#### 2.4.3. Experimental Evaluation of Coalescence: Hot-Stage Microscopy

Rheological measurements can give a preliminary indication of whether the polymers show a sufficient fluidity to successfully allow the coalescence of the molten particles; however, they do not simulate the coalescence process. This aspect can be further investigated using hot-stage microscopy (HSM). This technique combines optical microscopy and thermal analysis: a modern hot-stage microscope is typically composed of a computer-controlled programmable hot stage, where the sample is heated while placed normally on a glass slide; the events are recorded using a digital camera, and optical microscopy is employed for real-time observation [119].

**Figure 14:**
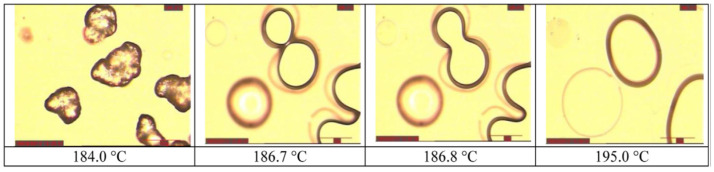
Evaluation of coalescence of PA12 particles using hot stage microscopy [97].

Typical images from HSM analyses taken from the study of Vasquez et al. [97] are shown in Figure 14. The evolution of several PA12 powder particles with the increase in temperature was observed and recorded. It is possible to follow the different steps of coalescence: from two separate particles to the formation of a neck between them, the growth of the neck, and finally the formation of a unique particle from the coalescence of the original particles.

As stated previously, hot-stage microscopy can be a useful tool to simulate the mechanisms of coalescence involved in the process of SLS; to best reproduce the process, the parameters of the hot stage should be set consequently. Laumer et al. [120] reported that the heating rates involved in SLS process could reach values of 10^7^ K/min; these values are nearly impossible to reproduce using any calorimetry technique. Nevertheless, an approach that was adopted in several studies was to configure the hot-stage program in two steps: the first was a fast heating to reach the sintering temperature, which was then followed by an isothermal step at these conditions. One of the first studies to use this method was conducted by Bellehumeur et al. [111], who investigated the coalescence of HDPE, LDPE, and propylene–ethylene copolymer powders in order to develop a model that considered the role of rheological properties on polymer sintering. The hot-stage program reported in this study included a first heating step that was used to reach the sintering temperature in approximately 15–20 s, although the heating rate was not specified. This was then followed by an isothermal step of 1200 s, in which the sintering sequence was recorded using time-lapse photography. Zhao et al. studied the coalescence of PA12 powder particles, aiming at verifying the validity of the previously mentioned sintering model proposed by Pokluda [110]. This work highlighted the dependence of the temperature at which coalescence began on the heating rate in the heating step: different temperatures of the onset of coalescence were measured with different heating rates, and the beginning of coalescence took place at higher temperatures with faster heating. In this way, it was possible to identify the correct temperature at which coalescence could occur during SLS [107]. Berretta et al. [116] studied the coalescence behavior of three different polymer powders belonging to the polyaryletherketone (PAEK) family, in particular polyether ether ketone (PEEK) and poly ether ketone (PEK) powders. The goal of the HSM analyses was to study particle coalescence under experimental conditions that could simulate the high-temperature laser sintering (HT-LS) process, in order to analyze the sintering behavior as a function of the viscosity of the molten powders. Two different runs were performed using HSM: the first analysis consisted of a heating step from room temperature to 400 °C at 120 °C/min; this step was carried out to identify the onset temperature of coalescence under high heating rates. A second test was performed by heating the samples from room temperature to 400 °C at 120 °C/min, and holding them at this temperature for 2 min, in order to best approximate the layer formation in HT-LS processing. The obtained results showed that the viscosity of the molten powders affected the rate of sintering, which was considered as the ratio between the neck length and the particles’ mean diameter. Another consideration concerned the different behavior of powders in the early phases of the process, which could be attributed to the particles morphology [116]. The coalescence of PAEK powders was also examined by Benedetti et al. [108], who used HSM to study the coalescence behavior of PEK and polyether ketone ketone (PEKK) powders. In this study, HSM analyses were performed from room temperature to 450 °C at a heating rate of 120 °C/min. The results showed that all powders exhibited shrinkage prior to melting, probably due to recovery of elastic deformation. Furthermore, this study also demonstrated the key role of viscosity in the process of coalescence, as well as the morphology of the particles, which influenced the particles’ melting, growth, coalescence, and final morphology.

Another approach that was adopted in several studies involving HSM was to use the same heating rate of DSC analyses, in order to compare the results obtained with these tests. Generally, DSC experiments are carried out with heating/cooling rates of 10 °C/min; it should be highlighted that these values are not comparable to the heating/cooling speeds involved in SLS process. In addition, the temperature at which the coalescence process took place was strongly linked to the applied heating rate, as previously cited [107]. Nevertheless, many studies have focused on the coalescence of polymer powders according to low heating rate, and so they should were considered in this review. Vasquez et al. [97] used HSM to study the coalescence behavior of PA12, TPE, and TPU powders, in order to identify differences in polymer sintering rates between those materials, with PA12 as the benchmark material in selective laser sintering. The curves of sintering showed a clear difference between PA12 and TPE and TPU, as the polyamide completed sintering in a much shorter time than the other two polymers. This could be attributed to a difference in viscosity between the polymers, the values of which, however, were not assessed. Verbelen et al. [112] studied the sintering behavior of different polyamides powders, specifically PA6, PA11, and two different grades of PA12 (with one of them showing a lower viscosity than the other). The results showed that PA11 and the PA12 with a lower viscosity rapidly coalesced to form a uniform film, while the more viscous PA12 and PA6 exhibited a slower coalescence, respectively due to the higher viscosity and the broad melting peak. In another study, Verbelen [121] investigated the sintering behavior of different TPU particles, showing good coalescence behavior due to low values of viscosity for all the investigated TPU grades. Moreover, coalescence took place in a much larger temperature range, if compared to conventional sintering polyamides; this was due to the different melting behaviors, which featured a much broader melting peak. Finally, Dadbakhsh et al. [106] investigated the coalescence behavior of virgin, aged, and mixed PA12 powders. The results of HSM analyses showed that coalescence took place in a narrower temperature range for the virgin PA12 powders (180–190 °C), while this range was broader for the mixed and aged powders. These results were in good agreement with rheological measurements, as it was shown that aged and mixed PA12 exhibited higher values of viscosity due to postcondensation phenomena.

### 2.5. Solidification/Crystallization (10^1^ min)

During the SLS fabrication process, the laser beam provides the energy required to selectively melt the preheated polymer particles on a selected region of powder bed surface, and to ensure the connection to the lower layer.

Then, the subsequent consolidation phase involves the cooling of the melt to the building temperature; this is higher than the crystallization temperature, which means that the powder melt does not immediately reach its final microstructure at this stage. In addition, according to the established model of a quasi-isotherm laser sintering [122], the melt pool and solid powder coexisted [99,123]: the liquid phase made by the molten powder and the solid phase, which referred to the surrounding powder, showed approximately the same temperature of the molten material.

In fact, the locally focused laser energy, in combination with the high melting enthalpy and the low thermal conductivity of the polymers, could hinder the fusion of surrounding powder particles [124]. This unmelted powder acted as both a support, ensuring the stability of the part during the building stage, and as an insulating layer, guaranteeing a uniform cooling and a homogeneous crystallization. When all layers were consolidated and stacked, the building chamber, including the unexposed material and the final component, cooled down to the room temperature, when it was then possible to extract the solidified part.

A detailed investigation of the crystallization/solidification behavior of polymer material and the mutual interaction with surrounding unmolten powder is required, due to their importance in the SLS process and their influence on built parts’ properties.

An investigation performed by Drummer et al. [123] compared the thermal behavior of various commercially available SLS powders such as PA12, POM (poloxymethylene), HDPE (high-density polyethylene), PP (polypropylene), and PEEK (polyether ether ketone).

As previously mentioned, the control of building temperature during SLS is a key factor in the process. Semicrystalline materials are in fact heated at a temperature that is higher than the glass transition point, and close to the melting point. In addition, the crystallization temperature must be significantly lower with respect to the melting temperature. In order to investigate the melting and crystallization behavior of the previously listed materials, DSC measurements were performed to simulate the SLS process. Different heating/cooling rates of 10, 5, and 1 K/min were applied. The results showed that the crystallization began at higher temperature when a lower cooling rate was considered. Since selective laser sintering is a slow process, a low cooling rate should be involved. This implies that during the SLS process, the crystallization can occur before the crystallization temperature, which is generally determined using a standard heating/cooling rate of 10 or 20 k/min.

In addition to the previously discussed measurements, DSC tests involving heating/cooling runs followed by an isothermal step at various temperatures higher than the crystallization onset were also performed. According to the quasi-isotherm model, the melt did not crystallize for a long period when a temperature lower than its melting point was maintained. Nevertheless, it was found that the peak of crystallization could significantly vary as a function of both time and temperature. Among the materials under investigation, PA12 is the most suitable for SLS process conditions, because it shows the crystallization peak after the longest isothermal time.

Further investigations of the crystallization and solidification phenomena led some authors to criticize the validity of the isothermal laser sintering model, and to put in evidence that its validity was limited to a narrow building time and, as a consequence, to a specified building height.

Amado et al. [125] performed an isothermal and a nonisothermal characterization of two SLS powders, polyamide 12 and co-polypropylene, respectively. A model for the determination of crystallization rate constant for the two powdered systems was elaborated; the obtained results were then used to perform an FEM simulation, inherent to the formation of the first 10 layers in a SLS job. The results showed a “stairs-effect” for each layer, which corresponded to a sequence of nonisothermal and quasi-isothermal states, in which the degree of crystallization could vary. Concerning the PA12, this variation was more significant: the crystallization degree was observed to change from 24% for the first layer up to 33% when 10th layer was deposited. Differently, PP instead showed a degree of crystallization of 12%, which did not significantly change for the subsequent layers.

In addition, the investigations performed by Drummer et al. [6] questioned the proposed isothermal laser sintering model, limiting its validity to the uppermost formed layers. The results showed that material crystallization could be observed in few layers under the powder bed surface; therefore, it occurred during the building process. This implied that the crystallization had already begun at the building temperature under quasi-isothermal conditions. As an alternative approach, the authors proposed an isothermal laser sintering process that described the progressive solidification of part during the building process.

Based on the same considerations that crystallization and then final consolidation of polymer powder began in the few layers underlying the bed surface, Greiner et al. [126] developed a new SLS process strategy for PA12 that involved a carefully monitoring and control of the temperature distribution in order to speed up the cooling in the building direction. With the aim to obtain similar starting conditions for a uniform cooling of each layer, an exposure strategy that was not dependent on the geometry and laser parameters was developed. Then, a dynamic cooling strategy with segmented temperature zones made possible an isothermal crystallization and a controlled cooling temperature gradient along the z-direction.

Because of the importance of the crystallization and solidification phases during the SLS process, authors began focusing their attention on studies based on crystallization kinetics and simulation of the material states as a function of temperature and time during the building and cooling steps.

It is well known that crystallization kinetics describe how the degree of crystallization changes as a function of both time and temperature, in which the degree of crystallization refers to the material volume changing from the molten to the solid state [10]. Zhao et al. [127] systematically investigated the crystallization behavior of PA12 powder, the most common material processed by SLS, through isothermal and nonisothermal DSC measurements. From the isothermal crystallization analysis, it was observed that a higher isothermal temperature led to a longer time for crystallization. Moreover, at a temperature similar to that used during the building process, crystallization was observed to occur after 50 min. This confirmed that layers lower than the exposed ones already began crystallizing during the building process. From nonisothermal crystallization measurements of PA12, it emerged that when using a smaller cooling rate, the onset of crystallization was recorded at a higher temperature, and crystallization occurred with a longer duration.

Moreover, the knowledge of crystallization kinetics, in addition to the possibility of monitoring the temperature profile for the parts that are forming, makes it possible to predict the crystallization behavior of each part as a function of its position on the building platform.

Thanks to the increasing interest in the development of new thermoplastic materials for selective laser sintering, similar studies were found in the literature for materials such as polypropylene [125,128,129] and polyetherketone [114,130].

#### Influence of Crystallization on Part Shrinkage and Deformation

In order to better understand the role of crystallization and solidification phenomena in the SLS process, the investigation of the crystallization kinetics must be performed in combination with the study of the factors that influence both the events and their effects on the properties of final components. In fact, according to Amado et al. [125], both the crystallization and solidification behavior are critical factors that affect the quality of the final parts in terms of density degree, deformation, and the presence of microstructural defects.

It is well known that shrinkage and warping are two critical issues that negatively affect the dimensional accuracy of parts processed by selective laser sintering [65]. Several researchers have attempted to control the entity of shrinkage, starting with the investigation of the different factors that influence it. Negi et al. [131] studied the effect of variation of SLS process parameters, revealing that the bed temperature, the hatching distance, and the scanning speed had the greatest impact on the shrinkage.

In addition, many studies identified crystallization as the main factor that caused the shrinkage, making it difficult to control the dimensional accuracy of the parts. It is worth noting that during the building process, a semicrystalline polymer underwent a volumetric change due to powder melting, solidification, and crystallization. The latter involved the rearrangement of molecule chains into a structure characterized by a lower volume. This change in volume and the resultant shrinkage were different as a function of the crystalline structure of semicrystalline polymer [112,132,133].

As an example, Van den Eynde at al. [134] reported a shrinkage of about 2.9% for LS-grade polybutene-1 in a tetragonal crystal structure, while a value more than double was observed for the same material in hexagonal polymorphic form.

Another factor that must be taken into consideration is the crystallinity degree: the higher the crystalline fraction of polymer, the higher the shrinkage that can be observed. This was confirmed by Shi et al. [55], who evaluated the shrinkage ratio of various commercial PA12 powders showing different crystalline degrees. The results showed that a high crystallinity of the material made it difficult to obtain a highly precise SLS component. The evaluation of the warp angle and the analysis of the DSC curves of the material during the melting–crystallization events further supported this outcome. Moreover, it was observed that when the melting and crystallization phenomena resulted in very separate peaks, the warping of the SLS part was not significant. On the contrary, when the two peaks were very close, the molten material turned very quickly into a solid and crystalline state without allowing a relaxation of stresses that cause warping.

In addition, Verbelen et al. [112], who studied how to vary the properties of different polyamide-based powders during the steps characteristic of the SLS process, reported that the determination of the temperature range between the melting and crystallization peaks was a good indicator to predict the tendency of a material to warp when solidified. Moreover, the study underlined that the crystallization shrinkage was greatly influenced by the crystallinity degree obtained upon cooling.

Benedetti et al. [135] considered three factors that induced shrinkage, and quantified their contributions: thermal retraction, recrystallization, and powder densification. The impact of these factors was found to be different as a function of the investigated material and the properties of the starting powders. For PA12, the shrinkage was mainly influenced by crystallization, while PEEK showed a lower dependence on this parameter. For this material, the powder bulk, a parameter describing the starting powder in term of morphology, porosity, and size distribution, showed the greatest impact on material shrinkage.

Most of the literature used experimental techniques to investigate the shrinkage and the factors influencing it; however, this approach showed some disadvantages, such as the cost and time consumption, and that it did not allow the researchers to distinguish the impact of each factor. On the basis of these considerations, Li et al. [65] deeply investigated the mechanism of shrinkage and warping by developing a numerical model supported by experimental data that took into account thermomechanical issues, heat transfer, and crystallization kinetics involved during the SLS process. It was found that the cooling rate greatly influenced the shrinkage: a high cooling rate implied an increment of crystallinity; the compaction of polymer molecular chains induced by crystallization of a material with increased crystalline fraction led to a high tendency of the material to shrink and warp.

## 3. Conclusions and Future Perspectives

Selective laser sintering is one of the most commonly used powder bed fusion technologies for the processing of polymers. Starting from a CAD model, the process involves the deposition of a homogeneous powder layer, and the use of laser radiation to selectively melt the powder bed. These steps are then repeated up to obtain the three-dimensional component. The SLS technique allows the manufacture of parts with a high dimensional accuracy, a high degree of customization, and good mechanical properties. These advantages make SLS a promising technique in many application fields, such as automotive, aerospace, and biomedical, for the production of functional prototypes, spare parts, and small series of components.

Although SLS seems to be the result of repeated simple steps, it involves different and quite complex physical phenomena that must be deeply investigated to optimize the process and to obtain high-performance components.

The present review described and discussed the complex physical phenomena involved during the SLS processing of polymers.

### 3.1. Powder Spreading

After the powder bed is preheated up to the powder bed temperature (T_b_), which is maintained during the whole process, a spreader is used to deposit a fixed amount of powder. The formation of a continuous, defect-free, and homogenous layer is fundamental to obtain a dense and high-performance component. The quality of the deposited layer is strongly affected by different parameters. Firstly, the properties of the starting powder, such as its particle size and distribution, as well as its morphology, play a key role, because they affect the flowability of the powdered system. The shape of the recoater profile is also important: the use of a roller allows the optimization of the interaction between the recoater and the powder, favoring the progressive rearrangement of particles. Moreover, the dynamics of the powder, involving the study of the mechanisms and forces by which the powder interacts with the spreader during the deposition step, is a critical issue.

### 3.2. Laser Motion and Irradiation

In SLS, the optical energy of the laser beam is converted into heat to selectively melt the material. Although it has been demonstrated that absorptance of polymeric powders at CO_2_ laser wavelength is high, the multiple scattering mechanism and the attenuation of the incident radiation through the powder bed depth greatly affect the optical-to-thermal energy conversion and the formation of melt pools in the scanned area. Different experimental and modeling techniques have been proved valuable to describe the complex phenomena involved in the laser–powder interaction. However, a database of the optical properties of polymeric powders, as well as a thorough understanding of the effect of fillers, is still missing. Therefore, the authors suggest that further research in these areas could be useful to bridge this gap, thus improving the prediction and optimization of energy-deposition parameters in SLS, and ultimately improving part properties.

### 3.3. Thermal Diffusion

Thermal diffusion phenomena govern the formation and the dynamics of the melt pool, which is formed when the laser beam provides the energy to selectively melt the powder bed; these phenomena are related to both the process parameters and the thermophysical properties of the polymer. A good understanding of the characteristics of the melt pool, with reference to its dimensions and the distribution of temperature, is necessary in a feasible process. For this reason, a great number of studies have focused on the investigation of the dynamics of the melt pool formation, using both numerical approaches and the evaluation of experimental results. Generally, these works studied the features of the melt pool as a function of process-parameter variation, and specifically of the energy density, which includes all the most relevant parameters involved in the laser sintering process.

### 3.4. Polymer Viscous Flow and Particle Coalescence

Viscous motion is the main driving force of consolidation phenomena that take place in selective laser sintering. In all the numerical models that were proposed to describe the viscous coalescence, zero-shear viscosity (which describes the fluidity of the polymer melt) was the quantity that most influenced the coalescence rate. For this reason, several studies focused on the evaluation of viscosity in the conditions occurring in selective laser sintering. Viscosity must show low values in order to provide high fluidity to the molten particles and to complete consolidation of the particles; this was experimentally confirmed by hot-stage microscopy, which can simulate the coalescence under conditions close to those occurring during SLS process.

### 3.5. Solidification/Crystallization

The formation of each layer is completed by the cooling of the melt to the building temperature, which is higher than that of the polymer crystallization. It is necessary to consider that when the laser locally provides the energy to melt a selected region of the powder bed, the high melting enthalpy and the low thermal conductivity of the polymer avoid the melting of surrounding powder particles. The understanding of the crystallization/solidification phenomena, and the interaction between the solidifying powder and surrounding unmolten particles is essential, because they affect the quality of the built parts in term of density, dimensional accuracy (shrinkage and deformation phenomena can occur), and the presence of microstructural defects.

Critical issues such as the crystallization kinetics and the simulation of the material development as a function of both temperature and time during the building and cooling process were discussed.

The increasing interest of both the scientific and industrial communities in selective laser sintering technology favors the continuous development of this process. The even more pressing demand for new materials to be processed by SLS is surely one of the most important and, at the same time, most critical issues that need to be addressed. This involves the introduction into the market of additional polymers that are suitable to be processed with this technology. In fact, some semicrystalline commodity polymers such as polypropylene (PP) and polyethylene (PE) find numerous applications in different fields such as engineering, packaging, and medical; however, their processability by SLS requires further investigations in order to be optimized.

In addition, the use of a reinforcement phase that can significantly increase the properties of the available materials is a promising approach to enlarge the SLS feedstock. When a secondary phase is introduced into a polymer powder, the complexity of the physical phenomena dramatically increases, and the previously described approaches must be improved. One of the most significant challenges is the understanding of how the SLS process is influenced by a polymer-composite powdered system. Because of the high potential impact of this issue, increasing efforts already have been made; however, further investigations are required.

## Data Availability

Data sharing not applicable. No new data were created or analyzed in this study. Data sharing is not applicable to this article.
